# Determinants for participation in a public health insurance program among residents of urban slums in Nairobi, Kenya: results from a cross-sectional survey

**DOI:** 10.1186/1472-6963-12-66

**Published:** 2012-03-19

**Authors:** James K Kimani, Remare Ettarh, Catherine Kyobutungi, Blessing Mberu, Kanyiva Muindi

**Affiliations:** 1 African Population and Health Research Center, APHRC Campus, 2nd Flr, Manga Close, Off Kirawa Road, P O Box 10787, 00100 Nairobi, Kenya

**Keywords:** Public health insurance, National Hospital Insurance Fund, Microfinance institutions, Urban slums, Kenya

## Abstract

**Background:**

The government of Kenya is making plans to implement a social health insurance program by transforming the National Hospital Insurance Fund (NHIF) into a universal health coverage program. This paper examines the determinants associated with participation in the NHIF among residents of urban slums in Nairobi city.

**Methods:**

The study used data from the Nairobi Urban Health and Demographic Surveillance System in two slums in Nairobi city, where a total of about 60,000 individuals living in approximately 23,000 households are under surveillance. Descriptive statistics and multivariate logistic regression analysis were used to describe the characteristics of the sample and to identify factors associated with participation in the NHIF program.

**Results:**

Only 10% of the respondents were participating in the NHIF program, while less than 1% (0.8%) had private insurance coverage. The majority of the respondents (89%) did not have any type of insurance coverage. Females were more likely to participate in the NHIF program (OR = 2.4; *p *< 0.001), while respondents who were formerly in a union (OR = 0.5; *p *< 0.05) and who were never in a union (OR = 0.6; *p *< 0.05) were less likely to have public insurance coverage. Respondents working in the formal employment sector (OR = 4.1; *p *< 0.001) were more likely to be enrolled in the NHIF program compared to those in the informal sector. Membership in microfinance institutions such as savings and credit cooperative organizations (SACCOs) and community-based savings and credit groups were important determinants of access to health insurance.

**Conclusions:**

The proportion of slum residents without any type of insurance is high, which underscores the need for a social health insurance program to ensure equitable access to health care among the poor and vulnerable segments of the population. As the Kenyan government moves toward transforming the NHIF into a universal health program, it is important to harness the unique opportunities offered by both the formal and informal microfinance institutions in improving health care capacity by considering them as viable financing options within a comprehensive national health financing policy framework.

## Background

Social health protection systems are mechanisms that countries use to address the challenges related to providing access to health care services to their citizens, especially the poor segments of the population. The benefits of extending social protection in health include reducing financial barriers associated with access to health care services and protection from financial catastrophe and impoverishment related to health care expenditures [1-5]. One of the categories of social health protection systems is the social health insurance, which is a financing scheme where monies are pooled into a common fund and used for paying for healthcare costs of members. Contributions are usually collected from workers, self-employed individuals, businesses and in some cases the government, particularly where a universal coverage model is adopted [1,3]. In Kenya, a universal social health insurance scheme has not been implemented; however, in November 2004, the government introduced the National Social Health Insurance Fund (NSHIF) Bill in parliament. The Bill was passed by parliament in December 2004 [6], but the President declined to assent to the Bill and sent it back to parliament due to a number of concerns. One of the concerns was that the Bill was deemed too expensive to implement and financially unsustainable [7]. As the government prepares to re-introduce the NSHIF legislation in parliament, it is important to have a better understanding of factors associated with participation in the current National Hospital Insurance Fund (NHIF), particularly among the poor, as well as a determination of the proportion of individuals without access to health insurance among this demographic group. The NSHIF will build on the existing NHIF framework and, therefore, such evidence is imperative in order to implement an effective NSHIF. It is hoped that the proposed NSHIF will have mechanisms that will increase equity and access to health care services by all population groups. One of the possible design features of the proposed NSHIF could be the implementation of flexible payment modalities and differentiated premium levels that will be deemed convenient to different socio-economic groups. This is important especially for individuals working in the informal sector, which is characterized by low and non-regular incomes [8]. With regard to payments collection, while premiums for those working in the formal sector can be collected through payroll deductions, one of the challenges faced by many African countries in the implementation of social health insurance schemes is how to come up with modalities for collecting contributions from the large proportion of the population working in the informal sector [3]. In this regard, the proposed NSHIF could institute mechanisms that can facilitate the collection of premiums from the informal sector through organized entities, such as the savings and credit cooperative organizations and community-based associations (e.g., farmers cooperatives, taxi and matatu (public transport vehicles) associations, and women's groups). Our study seeks to provide answers to the following research question: what are the factors associated with participation in the NHIF program among residents of urban slums?

Literature focusing on the determinants of participation in health insurance schemes in Kenya and Africa in general is limited. Studies conducted in a number of sub-Saharan African countries showed that employment in the formal sector was significantly associated with access to health insurance relative to being employed in the informal sector [2,3,9]. The low participation of individuals in the informal sector was attributed to a number of factors, including low and non-regular incomes, insecure employment, and insurance scheme design features (e.g., inflexible payment schedules and lack of awareness about insurance schemes) that are not adapted to people's needs and preferences. In Kenya, it is estimated that 31.6% and 26.3% of the total workforce are engaged in the informal and formal sectors, respectively, while 42.1% are engaged in small-scale farming and pastoralist activities [10]. Existing evidence also shows that membership in both formal (e.g., microfinance institutions such as the savings and credit cooperative organizations-SACCOs) and informal (e.g., community-based savings groups-popularly known as merry-go-rounds) savings and credit schemes, is an important predictor of participation in health insurance programs. According to previous studies, the saving and sharing mechanisms associated with these formal and informal schemes have provided the members with an opportunity to raise and collect funds to be used for various purposes, including payment of insurance premiums and addressing emergencies such as hospital care or funeral costs [2,11-13]. The formal savings and credit schemes comprise entities such as SACCOs that enable their members to save their money and also access loans. Membership is voluntary and members are offered a broad range of loan and savings products usually at a cheaper cost and flexible payment plans compared to mainstream commercial banking institutions. SACCOs predominantly provide access to financial services to people (i.e. poor and low-income groups) who would otherwise be excluded from accessing such services by mainstream banking institutions. These entities have formalized structures of management and are subject to government regulatory mechanisms [14-18]. Informal schemes (e.g., community-based savings groups-popularly known as merry-go-rounds) are formed on the basis of group solidarity mechanism where community members with common interests come together and pool funds by way of making contributions, which are then shared on a revolving basis. Each member receives the entire funds of the group after a set period of time [19]. A study on extending social health insurance to the informal sector in Kenya found that in the slums, informal savings groups were common and slum dwellers made monthly contributions, which were then shared on a revolving basis to each member of the group to help them cater for various needs, including funeral and health care costs [2]. Since these groups are not regulated by any formal legal mechanisms, the members depend on good faith and personal commitment and responsibility to ensure that individuals honor their obligations to contribute to the savings scheme. Other factors that were cited as key determinants of access to health insurance, included ownership of household assets such as land and livestock, higher income and education levels, and provision of social security and welfare services [20-23].

## Background on health insurance in Kenya

According to the Kenya National Bureau of Statistics, at the end of 2009, the total population in Kenya was 38.6 million [24]. In Kenya, more than four out of 10 (46.6%) individuals live below the poverty line [25]. Data from the national health accounts show that more than a third of the poor who were ill did not seek care compared to only 15% of the rich [26]. Additionally, according to the 2005/06 national health accounts, 36% of funds to the health sector came from households and out of these, the out-of-pocket expenditure accounted for more than 29% [27]. These findings raise concern about equity and financial accessibility to health care by a majority of people in Kenya, particularly the poor who are highly vulnerable to economic shocks that result from catastrophic out-of-pocket health expenditure. Existing studies show that the poor are more likely to get sick, less likely to use preventive and curative health care, and consequently, have higher mortality rates. According to these studies, one of the factors responsible for these challenges is high out-of-pocket payments for health care [28-30]. The 2010 World Health Report and the 2010 Millennium Development Goals report underscore the importance of reducing disparities in access to health care, particularly among the poor and marginalized groups through universal health coverage [31,32]. Extending access to health care to all segments of the population, including the poor is an important objective of the Kenyan government's national health sector strategic plan and national development agenda as outlined in the Kenya Vision 2030 policy framework [33-35].

The NSHIF legislation seeks to transform the current National Hospital Insurance Fund (NHIF) into a universal health coverage program, which will ensure equity and access to healthcare services by all citizens. One of the criticisms of the NHIF is its failure to reach out to the majority of Kenyans, especially the poor and those in the informal sector [2,3,9,36]. For example, the NHIF imposes a penalty that is five times the contribution amount for those who do not make their payments by the due date. This regulation particularly hurts the poor, the unemployed and casual workers in the informal sector, who do not have a steady income that would enable them, pay their contributions regularly. The NHIF was established by an Act of Parliament in 1966, as a national contributory hospital insurance scheme with the objective of providing Kenyan citizens with access to quality and affordable healthcare [37]. Contributions and membership are compulsory for all civil servants and formal sector employees, and voluntary for those in the informal sector and retirees. Members under the voluntary category pay a flat rate of Kshs 300 per month (approx. USD 4.00). For those in formal employment, contributions are made based on their income, but usually range between Kshs 150 to Kshs 2,000 per month (approx. USD 2.00 to USD 24.00). Currently, the NHIF only pays for inpatient costs at selected (mostly government) hospitals. Recently, a pilot outpatient service program was launched that allows members to get treatment in selected hospitals without having to be admitted. The NHIF is the most widely available medical cover in the country, with more than 400 accredited hospitals across the country, including government, faith-based and private ones. Copayment levels usually vary across hospitals, but generally fall under three types of contract options [38,39]. The first option applies to primarily government health facilities and beneficiaries' accessing services in these settings are comprehensively covered. The second option includes faith-based hospitals and some private hospitals (mostly in rural areas), where NHIF members also have comprehensive coverage but surgery costs are not covered. The surgery costs are covered on a copayment basis and payments are usually based on a capped amount. Finally, the third option focuses on medical services offered by high-cost private hospitals, where the NHIF provides a daily rebate for hospitalization which ranges from Kshs 400 to 2,000 per day (1 USD = 85 Kshs (May, 2011)) for a maximum of 180 days per beneficiary per year members. Any costs above this amount have to be borne directly by the beneficiary. Besides covering the principal member, the NHIF program also covers the principal's dependants, including the spouse and children (under and over 18 years). Nationally, in 2010, an estimated 2 million primary contributors and about 8 million dependants were enrolled in the NHIF program, with a majority (about 74%) residing in the urban areas [40]. Besides the NHIF, in Kenya, individuals can access health insurance through private insurance firms and to some extent community-based health insurance (CBHI) organizations. Due to cost considerations, private health insurance is predominantly accessible to the middle and higher-income groups [9]. Community-based health insurance is relatively new in Kenya having been established in 1999, and, as a result it has limited coverage [2]. According to the Kenya Community-Based Health Financing Association (KCBHFA), currently, there are nine institutions offering community health financing schemes with 410,997 beneficiaries or about 1% of the population covered [41]. In Africa, countries such as Burkina Faso, Senegal, Tanzania and Ghana have well developed CBHI schemes that are recognized by the national governments as a key component in the national health financing strategy [42-48]. Findings from these studies suggest that CBHI schemes have the ability to reach marginalized population groups such as the poor, women and children, however, more support and strategies from governments are needed to enhance their development and sustainability.

The objective of this paper is to assess the factors associated with participation in a public health insurance program (Kenyan National Hospital Insurance Fund-NHIF) among residents of urban slums in Nairobi city. In addition, this study seeks to determine the proportion of people without health insurance coverage in the slums of Nairobi. The findings from this study will provide important evidence with regard to the transformation of the NHIF into a universal health insurance scheme, which aims to grant all population groups, including the poor, access to quality and affordable health care services.

## Methods

### Study population and sampling

The data used in this paper came from the African Population & Health Research Center's (APHRC) project on Urbanization, Poverty and Health Dynamics (UPHD) in Nairobi. The broad aim of the UPHD project was to investigate the health consequences of rapid urbanization and growing urban poverty at different stages of the life course namely childhood, adolescence, adulthood, and old age. This research project is nested in the Nairobi Urban Health and Demographic Surveillance System (NUHDSS), which is run by APHRC in Viwandani and Korogocho slum settlements since 2002. These slum settlements, like most others in Nairobi are where over 60% of Nairobi's population live and are characterized by extreme poverty, poor access to health facilities, high morbidity and mortality rates, high unemployment rates and low school participation [49,50]. The NUHDSS covers a total of about 60,000 individuals living in approximately 23,000 households. For this analysis, we used cross-sectional data from the migration histories survey which were collected in 2006-2007 from a random sample of over 16,400 individuals above the age of 12 years drawn from the NUHDSS database. The migration histories survey, which was part of the UPHD project, aimed at documenting information on the inter-linkages between migration, poverty and health outcomes at the different stages of the life course.

### Data analysis

Descriptive, bivariate and multivariate analyses were carried out using STATA ^® ^version 10. The descriptive and bivariate analyses were used to describe the characteristics of the sample, including identifying the proportion of people without health insurance coverage in the slums and exploring the associations between the dependent variable (which is enrollment in the NHIF program) and the key independent (type of employment sector, participation in welfare programs, membership in savings and credit cooperative organizations and community-based savings groups) and other independent variables. For the bivariate analysis, chi-square test (*X*^2^) was used to test the association between enrollment in the NHIF and the explanatory variables. For the univariate regression analysis, the variables that were significant at α = 0.1 were selected and included in the multivariate analysis. The multivariate logistic regression analysis was conducted to examine the determinants for participation in the public health insurance program. Table 1 shows a summary of the variables and how they were operationalized. The dependent variable was participation in the public health insurance program. The key independent variables included in the multivariate analysis were the type of employment sector (formal and informal), participation in the National Social Security Fund (NSSF), participation in savings and credit cooperative organizations (SACCOs), and membership in community-based savings groups. Other independent variables that were controlled for in the regression analysis included income (monetary income only), gender, marital status, age, education, slum of residence, and ethnicity.

**Table 1:** Definition of variables in the analysis

Variable	Operational definition
**Dependent variable**	

Enrolment in NHIF	Yes = 1 and No = 0

**Key independent variables**	

Type of employment sector	1 = Formal sector and 0 = Informal sector

Participation in the National Social Security Fund (NSSF)	Yes = 1 and No = 0

Participation in savings and credit cooperative organizations (SACCOs)	Yes = 1 and No = 0

Participation in community-based savings groups	Yes = 1 and No = 0

**Other independent variables**	

Income in the last one month^a^	0 = Non-poor, 1 = Poor, and 2 = Don't know/missing

Gender	1 = Female and 0 = Men

Marital status	1 = Currently in union, 2 = Formerly in union, and 3 = Never in union

Age of respondents	0 = Less than 24 years, 1 = 24-29 years, 2 = 30-39 years, 3 = 40-49 years, and 4 = 50+ years

Education	0 = Never attended school, 1 = Primary, and 2 = Secondary/higher

Slum residence	0 = Viwandani and 1 = Korogocho

Ethnicity	0 = Kikuyu, 1 = Luhya, 2 = Luo, 3 = Kamba, and 4 = Other

**^a^**Individuals who earned less than USD 1.25 per day fell below the World Bank-defined poverty line and were categorized as poor, while those who earned this amount and above were defined as non-poor.

### Ethical considerations

The conduct of the Urbanization, Poverty and Health Dynamics project was approved by the Ethical Review Board of the Kenya Medical Research Institute (KEMRI). The field workers were trained in research ethics and obtained informed consent from all respondents. The NUHDSS has also been approved by KEMRI's Ethical Review Board.

## Results

### Descriptive analysis

The descriptive analysis demonstrated that a majority of the slum residents (89%) did not have any type of health insurance coverage, while only 10% had enrolled in the NHIF program (results not shown). Participation in private insurance schemes was very limited with less than one percent (0.8%) of the population in this category. As shown in Table 2, there was a significant association between being employed in the formal sector and enrolment in the NHIF. Nearly half of the respondents working in the formal sector (48%) and only 3% of those in the informal sector were enrolled in the NHIF program. A large majority (81%) of the respondents participating in the NSSF reported being members of the NHIF program. A significantly higher proportion of respondents who were members of the savings and credit cooperative organizations (SACCOs) and community-based savings, 90% and 21%, respectively, were enrolled in the NHIF program. With regard to income, a significantly lower proportion of the poor (only 2%) compared to individuals in the non-poor category (24%) were enrolled in the NHIF. Participation in the NHIF program was significantly higher among men (16%), among respondents who were currently in a union (16%), and among those with secondary school education and higher (20%).

**Table 2:** Study population characteristics and association between enrolment in NHIF and explanatory variables

		Enrolled in NHIF		
	
		Yes	No	
**Variables**	**Total Number**	**N (%)**	**N (%)**	***p*-values**

**Employment sector**				
Formal employment	3,007	1,434 (47.69)	1,573 (52.31)	***
Informal employment	6,000	180 (3.00)	2,113 (97.00)	
**Participates in National Social Security Fund (NSSF)**				
Yes	1,982	1,608 (81.13)	374 (18.87)	***
No	14,358	137 (0.95)	14,221 (99.05)	
**Participates in savings and credit cooperative organizations (SACCOs)**				
Yes	517	465 (89.94)	52 (10.06)	***
No	15,821	1,280 (8.09)	14,541 (91.91)	
**Participates in community-based savings groups**				
Yes	2,328	493 (21.18)	1,835 (78.82)	***
No	14,012	1,251 (8.93)	12,761 (91.07)	
**Income in the last one month**				
Poor	2,510	60 (2.39)	2,450 (97.61)	***
Non-poor	5,827	1,408 (24.16)	4,419(75.84)	
Don't know/missing	667	145 (21.74)	522 (78.26)	
**Gender**				
Male	8,699	1,413 (16.24)	7,286 (83.76)	***
Female	7,643	332 (4.34)	7,311 (95.66)	
**Marital status**				
Currently in union	7,399	1,190 (16.08)	6,209 (83.92)	***
Formerly in union	907	42 (4.63)	865 (95.37)	
Never in union	4,989	171 (3.43)	4,818 (96.57)	
**Age of respondents**				
Less than 24 years	3,992	45 (1.13)	3,947 (98.87)	***
24-29 years	3,096	177 (5.72)	2,919 (94.28)	
30-39 years	4,213	638 (15.14)	3,575 (84.86)	
40-49 years	2,174	437 (20.10)	1,737 (79.90)	
50+ years	2,867	448 (15.63)	2,419 (84.37)	
**Education**				
Never attended school	1,123	33 (2.94)	1,090 (97.06)	***
Primary	10,540	801 (7.60)	9,739 (92.40)	
Secondary/higher	4,327	871 (20.13)	3,456 (79.87)	
**Slum residence**				
Korogocho	7,741	207 (2.67)	7,534 (97.33)	***
Viwandani	8,601	1,538 (17.88)	7,063 (82.12)	
**Ethnicity**				
Kikuyu	5,535	347 (6.27)	5,188 (93.73)	***
Luhya	2,210	252 (11.40)	1,958 (88.60)	
Luo	2,394	142 (5.93)	2,252 (94.07)	
Kamba	3,871	756 (19.53)	3,115 (80.47)	
Other	2,302	247 (10.73)	2,055 (89.27)	

* *p *< 0.05; ** *p *< 0.01; *** *p *< 0.001; *X*^2 ^used to test the association between enrolment in the NHIF and explanatory variables

### Multivariate analysis

As presented in Table 3, being employed in the formal sector was significantly associated with a higher probability of having public health insurance (unadjusted odds ratio (UOR) = 29.47; *p *< 0.001 and adjusted odds ratio (AOR) = 4.11; *p *< 0.001, respectively) relative to working in the informal sector. Participation in the National Social Security Fund and membership in savings schemes such as SACCOs and community-based savings groups were significantly associated with having public health insurance. Income was an important predictor of health insurance ownership; specifically, respondents in the poor category were significantly less likely to be members of the NHIF compared to those in the non-poor category.

**Table 3:** Unadjusted and Adjusted odds ratios (ORs) and 95% confidence intervals (CIs) for determinants of public health insurance status

Variables	Public Health Insurance Status			
	
	Unadjusted OR (UOR)	95% CI	Adjusted OR (AOR)	95% CI
**Employment sector (*Ref = Informal employment)***				
Formal employment	29.47***	[25.00-34.75]	4.11***	[1.57-3.58]
Participates in National Social Security Fund (NSSF) ***(Ref = No)***	446.29***	[364.51-546.42]	222.63***	[152.34-325.35]
Participates in savings and credit cooperative organizations (SACCOs) ***(Ref = No)***	101.58***	[75.84-136.06]	14.48***	[7.48-28.03]
Participates in community-based savings groups ***(Ref = No)***	2.74***	[2.44-3.07]	1.62**	[1.15-2.28]
**Income in the last one month^a ^*(Ref = Non-poor)***				
Poor	0.07***	[0.05-0.09]	0.34***	[0.20-0.57]
**Gender *(Ref = Male)***				
Female	0.23***	[0.20-0.26]	2.37***	[1.57-3.58]
**Marital status *(Ref = Currently in Union)***				
Formerly in union	0.25***	[0.18-0.34]	0.46*	[0.23-0.94]
Never in union	0.18***	[0.15-0.21]	0.57*	[0.36-0.90]
**Age of respondents *(Ref = Less Than 24 Years)***				
24-29 years	5.31***	[3.82-7.40]	0.83	[0.24-2.82]
30-39 years	15.65***	[11.53-21.24]	0.76	[0.22-2.56]
40-49 years	22.06**	[16.15-30.14]	1.02	[0.29-3.54]
50+ years	16.24***	[11.90-22.16]	0.75	[0.21-2.63]
**Education *(Ref = Never Attended School)***				
Primary	2.71***	[1.90-3.86]	2.07	[0.88-4.86]
Secondary/higher	8.32***	[5.84-11.86]	2.12	[0.89-5.04]
**Slum residence *(Ref = Viwandani)***				
Korogocho	0.12***	[0.10-0.14]	0.84	[0.56-1.27]
**Ethnicity *(Ref = Other)***				
Kikuyu	1.92***	[1.62-2.28]	0.87	[0.55-1.38]
Luhya	0.94	[0.77-1.15]	1.67	[0.96-2.90]
Luo	3.62***	[3.17-4.15]	1.11	[0.77-1.62]
Kamba	1.79***	[1.51-2.13]	1.09	[0.68-1.76]

* *p *< 0.05; ** *p *< 0.01; *** *p *< 0.001. ^a^Cases with don't know/missing responses (n = 667) were excluded from analysis; UOR: analysis did not account for key confounding factors; AOR: adjustment for key confounders was done in the analysis

In the unadjusted regression analysis, women were less likely (UOR = 0.23; *p *< 0.001) to have been enrolled in the NHIF program compared to men, however, in the adjusted analysis they were more likely to have participated in the program (AOR = 2.37; *p *< 0.001). Marital status was also a significant predictor of having insurance coverage through the public health insurance program. Respondents who were formerly in a union (AOR = 0.46; *p *< 0.05) and who were never in a union (AOR = 0.57; *p *< 0.05) were less likely to participate in the NHIF program compared to those who were currently in a union. After controlling for other confounding factors, age, education, slum of residence, and ethnicity were not significantly associated with participation in the health insurance program.

## Discussion

The objective of this paper was to examine the determinants for participation in the Kenyan National Health Insurance Fund (NHIF) among urban slum dwellers. In addition, the study aimed at assessing the proportion of people without health insurance coverage. The findings show that a high proportion of slum residents (89%) have no access to any type of health insurance. With regard to participation in the NHIF, the data also indicate that fewer numbers of poor people and those from the informal sector are enrolled in the program. This finding corroborates evidence from previous studies, which demonstrated that the NHIF has not been effective in reaching out to the majority of Kenyans, especially the poor and those in the informal sector [2,9,36]. Interestingly, despite the fact that the NHIF is a compulsory scheme for formal sector employees, our study showed that less than half (48%) of the respondents from this sector were enrolled in the scheme. We could not find a plausible explanation for this observation and future research needs to investigate this outcome.

The multivariate results showed that a number of factors are significant predictors for participation in the public health insurance program, including employment in the formal sector, participation in social welfare programs (i.e., the national social security fund), participation in microfinance institutions and informal community-based savings groups', and being female. However, being poor, formerly in a union and never in a union were associated with a lower likelihood of participation in the public health insurance program. Similar to previous studies [2,3,9], our findings demonstrated that employment in the formal sector is an important determinant of participation in the NHIF program The differential participation in the NHIF between the formal and informal sectors has important implications on the expansion of social health insurance in Kenya. One objective of comprehensive social health insurance is to ensure that all population groups irrespective of their socio-economic status have access to quality and affordable health care. Since it is expected that the proposed NSHIF will benefit from the already established network of the NHIF program, our findings suggest that more efforts are needed to integrate informal sector workers into the NHIF. A study by Mathauer et al. to explore how to increase the participation of informal sector workers in the NHIF found that some of the design features of the program acted as critical barriers [2]. These included absence of flexible payment mechanisms, differentiated contribution amounts and punitive penalties for those who do not pay their contributions on time. These factors fail to take into account the low and non-regular incomes, which are a common phenomenon among workers employed in the informal sector [8]. Participation in the National Social Security Fund (NSSF) was also an important determinant for enrolling in the NHIF program. The NSSF is a public scheme that offers social protection to Kenyan workers against stoppage or considerable reduction of earnings due to a number of factors, including sickness and old age. The scheme covers workers in both formal and informal sectors, however, the former accounts for a large proportion of the scheme's membership. The amount of monthly contributions vary depending on a worker's wage or salary and benefits payable to eligible members are based on three levels-age/retirement benefit, withdrawal benefit and invalidity benefit [51]. In addition, there are also grants that are paid to members who are permanently emigrating from Kenya and dependants of deceased members. One plausible explanation of why participation in the NSSF was closely linked to the outcome variable is because similar to the NHIF, membership to the NSSF is compulsory for workers employed in the formal sector.

Our findings provide evidence on the potential of microfinance institutions (e.g., SACCOs) and informal community-based savings groups (popularly known as merry-go-rounds) as mechanisms through which funds can be pooled to assure access to health care for the poor/indigent segments of the population. These findings corroborate those of previous research, which found that membership in savings and credit associations and community-based savings groups provided individuals with a means of paying for health insurance contributions [2,11,12,22,23], hence increasing their access to health insurance. These entities enable group members to raise and collect funds to be used for emergency situations, such as paying for hospital or funeral costs. Based on our study findings, the two types' of microfinance schemes may be influencing participation in the NHIF through the saving mechanism, which allow members to access money for paying contributions to the scheme. In this respect, the microfinance institutions and community-based savings groups have an important role to play in the proposed NSHIF with regard to collection of contributions from members especially those from the informal sector. Unlike in the formal sector, it is difficult to assess incomes and collect income taxes from workers employed in the informal sector [9] and, as a consequence, deduction of contributions for the NSHIF can be a challenge. This means that lack of suitable mechanisms for colleting contributions from people employed in the informal sector could hamper the implementation and sustainability of the proposed social health insurance program. Previous research has shown that in many African countries, one of the challenges that have hindered efforts to implement comprehensive social health insurance is the difficulty of collecting contributions due to a high proportion of the population in the informal sector [3,9]. Evidence shows that many workers in the informal sector participate in SACCOs and community-based groups (e.g., merry-go-rounds) [19] and, therefore, these organized units can be important platforms through which contributions are collected and submitted to the NSHIF. Since the informal sector accounts for the largest proportion of Kenya's total workforce [10], the close linkage between the microfinance schemes and the informal sector can be leveraged and enhanced to enable the two types of schemes act as collection points for NSHIF contributions.

Similar to previous studies [2,3], our results also show that the poor were significantly less likely to participate in the NHIF program. This finding is indicative of the fact that the poor may not be able to pay the required contributions to the NHIF program. Women were more likely to participate in the program compared to men. However, in the univariate analysis the result was in the opposite direction. One plausible explanation is that women are more likely to be members of the microfinance and community-based savings mechanisms (commonly referred to as merry-go-rounds) and, therefore, controlling for this variable in the multivariate analysis increased the probability of women's participation in the NHIF program. Previous research has shown that women are more likely to be members of community-based saving groups compared to women [52]. Marital status was also an important predictor of participation in the NHIF. Respondents who were formerly in a union and those who were never in a union were less likely to participate in the public health insurance program relative to those who are currently in a union. A possible explanation for this finding is that when individuals get divorced, widowed or separated, they may become financially vulnerable hence impacting their ability to make payments to the NHIF program. Similarly, individuals who were never in a union may also experience financial constraints that limit their ability to make payments to the program. These findings suggest that having a spouse/partner is beneficial possibly because of the financial support derived from being in a dual-income household.

The findings from our study have two important policy implications. First, the large proportion of the urban poor without health insurance and the inadequate coverage of the poor by the NHIF highlights the need by the government to hasten the move towards social health protection by implementing a National Social Health Insurance Fund to guarantee access to quality healthcare services for the poor and vulnerable segments of the population, as well offer protection against financial shocks associated with high medical costs. The lack of insurance coverage among the urban poor exposes them to heavy financial burden associated with catastrophic out-of-pocket health expenditure. To ensure that the vulnerable and poor have access to health care under the NSHIF, the government will need to institute targeted subsidies and exemptions aimed at increasing their enrolment. Also, since the proposed NSHIF is expected to benefit from the networks and structures already established under the NHIF, therefore, there is need by the government to reach out to the informal sector workers. Our study shows that people employed in the informal sector are less likely to be enrolled in the NHIF program. Considering that the informal sector accounts for the highest proportion of Kenya's total workforce, reaching out to this sector is critical for the successful implementation of the social health insurance scheme. Secondly, there is need to harness the potential of microfinance and community-based savings mechanisms in addressing existing gaps in healthcare financing and accessibility. Existing data indicate that in Africa, Kenya has the third largest proportion of poor people (accounting for more than 8% of Kenya's poor people) who belong to a microfinance institution [13]. Consequently, among the poor segments of the population, research evidence shows that these mechanisms are instrumental in providing income-earning opportunities. Similarly, our study results suggest that the same platforms may also help the poor and underserved populations to access health services, particularly where they must pay for them. The government should strengthen the microfinance and community-based savings mechanisms and utilize them as platforms for collecting contributions from the informal sector. It is important to be cognizant of the fact that not all poor people belong to these microfinance and community-based savings mechanisms because they can not afford to put money aside as savings and, therefore, in such cases the government should make contributions on their behalf to the NSHIF.

## Limitations

Our study had a number of limitations that need to be highlighted. First, the data were from only two urban slums in Nairobi, and, therefore, the findings from our study are not generalizable to all the slum areas in Kenya. Second, due to the lack of data on respondents' health status (e.g., presence of illnesses, frequency of illnesses), we were unable to assess the association between health status and having health insurance coverage. Previous studies have shown that health status is an important predicator of health insurance coverage [23,45,48]. Also, no data were collected on out-of-pocket payments and health care utilization; therefore, it was not possible to examine the effect of having health insurance on these two outcomes.

## Conclusion

According to the United Nations, addressing the disparities in access to care among the poor and marginalized groups is critical in accelerating the achievement of the Millennium Development Goals (MDGs). The findings from this study highlight key issues with regard to access to health insurance among the urban poor. In this regard, the NHIF can explore ways of partnering with microfinance institutions to create mechanisms through which members of these institutions, particularly the poor and those in the informal sector can participate in the insurance program. Our study also showed that only a small proportion of the urban poor and those from the informal sector are enrolled in the NHIF program. Consequently, there is need to ramp up efforts aimed at increasing the enrollment of poor or financially challenged people in the program.

Existing evidence shows that efforts to implement social health insurance programs by many African countries, including Kenya are hampered by lack of sustainable health financing mechanisms. As the Kenyan government moves toward transforming the NHIF into a universal health coverage program, it is important to harness the unique opportunities presented by formal and informal microfinance platforms in improving health care capacity by considering them as viable financing options within a comprehensive national health financing policy framework. The government should hasten the plans to implement a universal health coverage program in order to facilitate improved access to quality and affordable health care.

## Conflict of interests

The authors declare that they have no competing interests.

## Authors' contributions

JKK conceptualized the study, conducted the data analyses, participated in the literature review, and prepared the first draft of the manuscript. RE made substantive contribution that informed the data analyses and reviewed the manuscript. CK made substantive contribution to the conceptualization of the study and reviewed the manuscript. BM and KM were involved in revising the manuscript for intellectual content and interpretation of data. All authors are aware that the manuscript is being submitted to the journal. All authors read and approved the final manuscript.

## Pre-publication history

The pre-publication history for this paper can be accessed here:

http://www.biomedcentral.com/1472-6963/12/66/prepub

## References

[B1] World Health Organization Paying for health services 2007 Geneva, Switzerland http://www.who.int/mediacentre/factsheets/fs320.pdf

[B2] Mathauer I Schmidt JO Wenyaa M Extending social health insurance to the informal sector in Kenya. An assessment of factors affecting demand Int J Heal Plan Manag 2008 23 51 68 10.1002/hpm.914 18050152

[B3] Kirigia JM Preker A Carrin G Mwikisa C Diarra-Nama AJ An overview of health financing patterns and the way forward in the WHO African region The East Afr Med J 2006 83 8 S1 S27 10.4314/eamj.v83i9.9492 17476860

[B4] Quayyum Z Nadjib M Ensor T Sucahya PK Expenditure on obstetric care and the protective effect of insurance on the poor: lessons from two Indonesian districts Health Policy Plan 2010 25 3 237 247 10.1093/heapol/czp060 20007133

[B5] Hidayat B Thabrany H Dong H Sauerborn R The effects of mandatory health insurance on equity in access to outpatient care in Indonesia Health Policy and Planning 2004 19 5 322 335 10.1093/heapol/czh037 15310667

[B6] World Health Organization Health financing reform in Kenya: assessing the social health insurance proposal 2006 Geneva, Switzerland: WHO, Department "Health System Financing" (HSF) Cluster "Evidence and Information for Policy" (EIP)

[B7] Hakijamii Trust (Economic and Social Rights Centre) The Right to Social Security in Kenya: The gap between international human rights and domestic law and policy Nairobi, Kenya 2007 http://www2.ohchr.org/english/bodies/cescr/docs/info-ngos/hakijamiikenya39.pdf

[B8] Xaba J Horn P Motala S The informal sector in Sub-Saharan Africa 2002 Geneva, Switzerland: Employment Sector, International Labour Office

[B9] Kimani D Muthaka DI Manda DK Healthcare Financing Through Health Insurance in Kenya. The Shift to A National Social Health Insurance Scheme 2004 Nairobi, Kenya: Kenya Institute for Public Policy Research and Analysis 1 71

[B10] Kenya National Bureau of Statistics Report of 1998/99 Labour Force Survey 2003 Nairobi, Kenya: Kenya National Bureau of Statistics

[B11] Dekker M Wilms A Health insurance and other risk-coping strategies in Uganda: the case of microcare insurance Ltd World Dev 2010 38 3 369 378 10.1016/j.worlddev.2009.09.004

[B12] Leatherman S Dunford C Linking health to microfinance to reduce poverty Bull World Health Organ 2010 88 470 47 10.2471/BLT.09.071464 20539866 PMC2878149

[B13] Moeller M Micro Insurance in Health: A summary of key outcomes of annual micro-finance conference - 2009 http://www.gtzkenyahealth.com/blog3/?p=3997

[B14] Sacco Societies Regulatory Authority The SACCO Socities Act: Kenya Subsidiary Legislation 2010 Legal Notice No 95. Volume Legislative Supplement No. 27 2010 Kenya: Kenya Union of Savings & Credit Co-operatives 343 461

[B15] German Cooperative and Raiffeisen Confederation The role of and importance of savings and credit cooperatives in microfinance and the worldwide activities of the German cooperative and raiffeisen confederation (DGRV) 2005 Bonn, Germany: International Relations Department, Deutscher Genossenschafts- und Raiffeisenverband e.V

[B16] Okwany A Grace Dudlu Transformative development: Harnessing the cooperative entrepreneurship advantage for women and youth in Africa The 11th SACCA Congress Meeting on 'fostering the Culture of entrepreneurship and Innovation in SACCOs' organized by Africa Confederation of Co-operative Saving and Credit Association (ACCOSCA) in collaboration with the Government of Swaziland, Swaziland Association of Savings & Credit Coop (SASCCO) and Canadian Co-operative Association 2010 Swaziland: Africa Confederation of Co-operative Saving and Credit Association (ACCOSCA) 1 12

[B17] World Bank Investments in Rural Finance for Agriculture Volume Module 8 2006 Washington, DC, USA: The World Bank

[B18] Kimani M Mobilizing money at the grass roots self-help groups in Kenya boost domestic savings and investment Africa Renewal 2009 23

[B19] Mputhia C Ochieng' Rapuro Benefits of legally registering investment clubs law Nation, Business Daily 2010 Business Daily edition. Nairobi, Kenya: Nation Media Group 1 3 http://www.businessdailyafrica.com/Benefits+of+legally+registering+investment+clubs+law/-/539444/952030/-/view/printVersion/-/k4yogkz/-/index.html

[B20] Onwujekwe O Okereke E Onoka C Uzochukwu B Kirigia J Petu A Willingness to pay for community-based health insurance in Nigeria: do economic status and place of residence matter? Health Policy and Planning 2010 25 155 161 10.1093/heapol/czp046 20156920

[B21] Perlman F Balabanova D McKee M An analysis of trends and determinants of health insurance and healthcare utilisation in the Russian population between 2000 and 2004: the 'inverse care law' in action BMC Heal Serv Res 2009 9 68 10.1186/1472-6963-9-68 PMC2684531 19397799

[B22] Russell S Ability to pay for health care: concepts and evidence Health Policy and Planning 1996 11 3 219 237 10.1093/heapol/11.3.219 10160370

[B23] Schneider P Why should the poor insure? Theories of decision-making in the context of health insurance Health Policy and Planning 2004 19 6 349 355 10.1093/heapol/czh050 15459160

[B24] Kenya National Bureau of Statistics Kenya 2009 Population and Housing Census Highlights 2010 Nairobi, Kenya: Kenya National Bureau of Statistics

[B25] World Bank Poverty data by country Washington, DC 2010 http://data.worldbank.org/country/kenya

[B26] Abt Associates I Kenya National Health Accounts 2002: Estimating Expenditures on General Health and HIV/AIDS Care 2005 Bethesda, Maryland, USA: Abt Associates, Inc.

[B27] Government of Kenya Health Systems 2020 Project Kenya National Health Accounts 2005/2006 2009 Bethesda, MD: Health Systems 20/20 project, Abt Associates Inc

[B28] Ziraba AK Samuel Mills Nyovani Madise Teresa Saliku Fotso J-C The state of emergency obstetric care services in Nairobi informal settlements and environs: Results from a maternity health facility survey BMC Heal Serv Res 2009 9 46 10.1186/1472-6963-9-46 PMC2662828 19284626

[B29] Perkins M Ellen Brazier Ellen Themmen Brahima Bassane Djeneba Diallo Angeline Mutunga Mwakajonga T Ngobola O Out-of-pocket costs for facility-based maternity care in three African countries Health Policy and Planning 2009 24 289 300 10.1093/heapol/czp013 19346273 PMC2699243

[B30] Falkingham J Akkazieva B Baschieri A Trends in out-of-pocket payments for health care in Kyrgyzstan, 2001-2007 Health Policy and Planning 2010 25 5 427 436 10.1093/heapol/czq011 20332252 PMC3072825

[B31] Evans DB Etienne C Health systems financing and the path to universal coverage Bull WHO 2010 88 402 20539847 10.2471/BLT.10.078741 PMC2878164

[B32] United Nations The Millennium Development Goals Report 2010. United Nations Department of Economic and Social Affairs (DESA) 2010 New York, U.S.A.: United Nations

[B33] Kenya Ministry of Medical Services Ministry of Medical Services Strategic Plan, 2008-2012 2008 Ministry of Medical Services

[B34] Kenya Ministry of Health Reversing the Trends: The Second National Health Sector Strategic Plan of Kenya - NHSSP II: Midterm Review Report. Ministry of Health - Sector Planning and Monitoring Department 2007

[B35] Ministry of Planning and National Development Vision 2030 2009 Nairobi: Ministry of Planning and National Development

[B36] Carrin G Chris James Michael Adelhardt Ole Doetinchem Peter Eriki Mohammed Hassan van den Hombergh Henri Joses Kirigia Burkard Koemm Rolf Korte Health financing reform in Kenya - assessing the social health insurance proposal South Africa Medical Journal 2007 97 2 130 135 17404675

[B37] Retirement Benefits Authority Historical background of the National Health Insurance Fund 2009 http://www.google.co.ke/url?sa=t&rct=j&q=nhif%20historical%20background%20of%20the%20national%20health%20insurance%20fund.%20national%20health%20insurance%20fund&source=web&cd=1&ved=0CCIQFjAA&url=http%3A%2F%2Fwww.rba.go.ke%2Fnews-and-events%2Fpresentations%2Fcategory%2F30-presentations-2009%3Fdownload%3D99%253Athe-social-health-insurer-of-choice--by-national-hospital-insurance-fund-nhif&ei=b8dVT42UMInVrQe5qoivBw&usg=AFQjCNHC-ucN4jf0VWixW2-iknbkI_N-7Q

[B38] Joint Learning Network on Universal Health Coverage Kenya: National Hospital Insurance Fund 2011 http://www.jointlearningnetwork.org/content/national-hospital-insurance-fund

[B39] Midiwo G Franziska Fuerst, Inke Mathauer Quality management actors and instruments and their institutional links to social health protection mechanisms Conference on Assuring Quality Health Care through Social Health Protection:The role of strategic purchasing and quality management: 2007 2007 Kigali, Rwanda 1 17

[B40] Government of Kenya Health Systems 2020 Project August 2010. Report of the Technical Working Group on Sustainability for the Kenya HIV/AIDS Program 2010 Bethesda, MD: Health Systems 20/20 project, Abt Associates Inc

[B41] The Role of KCBHFA in the Transformation of National Hospital Insurance Fund (NHIF) to National Social Health Insurance Fund (NSHIF) http://www.kcbhfa.org/training%20materials/role%20of%20kcbhfa%20in%20transformation%20of%20nhif.pdf

[B42] De Allegri M Sanon M Bridges J Sauerborn R Understanding consumers' preferences and decision to enrol in community-based health insurance in rural West Africa Health Policy 2006 76 58 71 10.1016/j.healthpol.2005.04.010 15946762

[B43] De Allegri M Sanon M Sauerborn R To enrol or not to enrol?": a qualitative investigation of demand for health insurance in rural West Africa Social Sci Med 2006 62 1520 1527 10.1016/j.socscimed.2005.07.036 16169645

[B44] Bennett S The role of community-based health insurance within the health care financing system: a framework for analysis Health Policy and Planning 2004 19 3 147 158 10.1093/heapol/czh018 15070863

[B45] Dong H De Allegri M Gnawali D Souares A Sauerborn R Drop-out analysis of community-based health insurance membership at Nouna, Burkina Faso Health Policy 2009 92 174 179 10.1016/j.healthpol.2009.03.013 19394105

[B46] Dong H Mugisha F Gbangouc A Kouyate B Sauerborn R The feasibility of community-based health insurance in Burkina Faso Health Policy 2004 69 45 53 10.1016/j.healthpol.2003.12.001 15484606

[B47] Franco LM Diop FP Burgert CR Kelley AG Makinena M Simparae CHT Effects of mutual health organizations on use of priority health-care services in urban and rural Mali: a case-control study Bull WHO 2008 86 830 838 19030688 10.2471/BLT.08.051045 PMC2649553

[B48] Jütting J Health insuarnce for the poor? 2003 OECD: Determinants of participation in community-based health insurance schemes in rural Senegal

[B49] APHRC Population and Health Dynamics in Nairobi's Informal Settlements: Report of the Nairobi Cross-sectional Slums Survey (NCSS) 2000 2002 Nairobi

[B50] UN-HABITAT Nairobi Urban Sector Profile 2006 Nairobi, Kenya: UN-HABITAT Regional Office for Africa and the Arab States

[B51] Chitembwe S Abdalla Bujra The role of NSSF in the welfare and development of the Kenyan society Mijadala on Social Policy, Governance and Development in Kenya 2007 Nairobi Safari Club, Nairobi, Kenya: Development Policy Management Forum 245 254

[B52] Kitetu C Organisational networks of Kenyan female migrants in England: The humble chama now operating at higher international levels

